# Segment and Recover: Defending Object Detectors Against Adversarial Patch Attacks

**DOI:** 10.3390/jimaging11090316

**Published:** 2025-09-15

**Authors:** Haotian Gu, Hamidreza Jafarnejadsani

**Affiliations:** Department of Mechanical Engineering, Stevens Institute of Technology, Hoboken, NJ 07030, USA; hgu8@stevens.edu

**Keywords:** patch-enabled image attack, adversarial robustness, object detection model, vision-based object tracking

## Abstract

Object detection is used to automatically identify and locate specific objects within images or videos for applications like autonomous driving, security surveillance, and medical imaging. Protecting object detection models against adversarial attacks, particularly malicious patches, is crucial to ensure reliable and safe performance in safety-critical applications, where misdetections can lead to severe consequences. Existing defenses against patch attacks are primarily designed for stationary scenes and struggle against adversarial image patches that vary in scale, position, and orientation in dynamic environments.In this paper, we introduce SAR, a patch-agnostic defense scheme based on image preprocessing that does not require additional model training. By integration of the patch-agnostic detection frontend with an additional broken pixel restoration backend, Segment and Recover (SAR) is developed for the large-mask-covered object-hiding attack. Our approach breaks the limitation of the patch scale, shape, and location, accurately localizes the adversarial patch on the frontend, and restores the broken pixel on the backend. Our evaluations of the clean performance demonstrate that SAR is compatible with a variety of pretrained object detectors. Moreover, SAR exhibits notable resilience improvements over state-of-the-art methods evaluated in this paper. Our comprehensive evaluation studies involve diverse patch types, such as localized-noise, printable, visible, and adaptive adversarial patches.

## 1. Introduction

Deep neural networks (DNNs) are increasingly deployed in the physical world for safety-critical computer vision tasks, such as face authentication on smartphones, driving assistance in autonomous cars, and intruder detection on surveillance cameras. However, DNNs are known to be vulnerable to evasion attacks, perturbations that when combined with data inputs, cause intentional misclassifications. It poses a serious threat to real-world object detection systems, since they are easy to implement physically. While there is an abundance of adversarial patch attacks on object detectors [1,2,3,4,5,6,7,8] and classifiers [9,10,11,12,13,14,15,16], defenses against such attacks have not been extensively studied. Securing object detectors is more challenging due to the complexity of the task. Defenses against adversarial patch attacks on object detectors can be mainly grouped into five types: (i) adversarial training, (ii) detection and removal [17,18,19,20,21,22,23], (iii) detection and mitigation [24,25,26,27], (iv) detection and restoration [28,29], and (v) certifiably robust defenses [30,31,32,33,34,35,36,37,38,39].

While adversarial patches are localized, they can affect predictions not only locally but also on objects that are farther away in the image because object detection algorithms utilize spatial context for reasoning. This effect is especially significant for deep learning models, as a small localized adversarial patch can greatly disturb feature maps on a large scale due to large receptive fields of neurons. Removing them after detecting from the images will lose object pixels for detection. Instead, restoring the broken pixels will minimize the adverse effects of adversarial patches, both locally and globally, on detection. In this paper, we present the Segment-and-Recover (SAR) defense, a “detect-and-restore” strategy-based framework, which can robustify any object detector against patch attacks without retraining the object detectors. (e.g., All of adversarial patch attacked target in Table 1 are identified by varied detector).

The main contributions of the paper can be condensed as follows:

- By integration of the patch-agnostic defense-based frontend with an additional broken pixel restoration backend, we developed Segment and Recover (SAR) for detecting adversarial image patches and recovering the object detection accuracy;
- We revealed adversarial patches in the high-frequency domain and proposed a recompression-based patch localization frontend, which is agnostic to patch appearance, shape, and location;
- We conducted extensive evaluations and comparative studies on state-of-the-art approaches for adversarial patches of varying sizes, tasks, and attack models. The results demonstrate that our method outperforms all the evaluated state-of-the-art approaches, particularly in terms of object detection accuracy.

## 2. Related Work

### 2.1. Adversarial Patch Attacks

Adversarial patch attacks were first introduced in [9]. It proposed universal, robust, targeted adversarial image patches to prevent classifiers [43,44,45,46] from identifying the target object in an image. This patch aims to find an input that maximizes the loss function. Using this loss function, the goal of the adversary is to directly minimize that probability until it falls below the detection threshold of the network. RP_2 [10], QR Patch [12], PS-GAN [13], DiAP [14], camera stickers [15], and EOT [16] are introduced to attack the classifier, but they are not able to fool object detectors such as Faster R-CNN [41] and YOLO [40]. The reason is that modern object detectors first locate objects of different sizes at different locations in the image and then perform classifications. DPATCH [1] is the first patch attack against object detectors, proposed as an iteratively trained adversarial patch that effectively attacks bounding box regression and object classification simultaneously. To include more training loss, EAVISE [47] creates an adversarial patch that is successfully able to hide persons from a person detector, where the optimization goal consists of the non-printability score, the maximum objectness score, and the total variation in the image. Instead of considering the non-printability score in the training loss, [48] adds the total patch saliency to train the adversarial patch. Some localized patches are introduced [2,4,6,7,8,49] to attack detectors such as YOLO and Faster R-CNN. Furthermore, a translucent patch [50] is proposed as a small opaque dot placed upon the camera lens. Assuming sufficient lighting, such adversary overlays can be well approximated by an alpha-blending operation between the original image and an appropriately sized and colored dot.

### 2.2. Defenses Against Patch Attacks

Generally, the defense methods can be categorized as detection-based and image-preprocessing-based methods. The detection-based method expects the detector to identify the adversarial risk while guaranteeing the detection accuracy of the target. The preprocessing-based method, which includes “Detect-and-Smooth”, “Detect-and-Remove”, and “Detect-and-Inpaint” strategies, expects to detect and post-process the adversary in images to defend against the visible attack.

Adversarial training introduces discovered adversarial examples and the corresponding ground-truth labels to the training. Ideally, the model will learn how to restore the ground truth from the adversarial perturbations and perform robustly on future adversarial examples. A recent study showed promising results for defending against patch attacks using adversarial training [51]. This technique, however, suffers from the high cost of generating adversarial examples and (at least) doubles the training cost of DNN models due to its iterative retraining procedure. Its effectiveness also depends on having a technique for efficiently generating adversarial examples similar to the one used by the adversary, which may not be the case in practice. As pointed out by Papernot in [52], it is essential to include adversarial examples produced by all the known attacks in adversarial training, since this defensive training is non-adaptive.

Some defense methods, such as detection and mitigation [24,25,26,27], propose to remove and refill the identified candidate regions using inpainting to mitigate the potential adverse effects. Local gradient smoothing (LGS [24] has been used to develop an effective method to estimate noise location in the gradient domain and transform those high-activation regions caused by adversarial noise in the image domain while having minimal effect on the salient object that is important for correct classification. In [27], signal-based feature extraction is used to “detect and mitigate” the adversarial patch. The first step is compressing the input image for error-level analysis (ELA) to identify the adjacent regions between the adversarial patch and the original image. However, accurately determining the key adversarial pixel is unrealistic because the adversarial gradient of the patch is unknown beforehand. Furthermore, if the patch covers too much area of the object, the refilled area contains too many pixels, which are far away from the original object. It will occlude the objects to be detected or classified.

To provide more general defense, researchers have explored locating patch areas and removing adversarial effects with a detect-and-remove-based strategy [17,18,19,20,21,22,53]. Hayes [17] proposed digital watermarking (DW), a “detect-and-remove” strategy with localized and visible adversarial perturbations to defend against adversarial patches for non-blind and blind image inpainting, inspired by the procedure of digital watermarking removal. In this method, a saliency map of the image was constructed to help remove small holes and mask the adversarial image, blocking adversarial perturbations. While Segment and Complete (SAC) [19] performs better for the PGD-generated adversarial patch [54], it struggles to defend natural-looking patches. Furthermore, SAC depends on the patch location and scale estimation. The PAD [20] is proposed for various adversarial patch localizations without relying on prior attack knowledge (e.g., appearance, shape, size, and quantity) and removal without additional training. Although the “removing” operation can eliminate the effect of the adversarial patches, it destroys the contextual information within the original image. Therefore, these incomplete images will affect the performances of downstream tasks, like image classification or object detection. Compared with this “detecting-and-removing” strategy, an alternative option should be a “detecting-and-recovering” strategy, i.e., recovering or repairing the original content covered by the adversarial patch after detecting the adversarial region. Jedi [28] and RLID [29] are proposed based on this strategy.

## 3. Problem Setup

In this section, we first introduce the image detector model, followed by the adversarial patch attack. Also, we present notations and terminology used in this paper. Table 2 provides a summary of the notations.

### 3.1. Image Object Detector

Object classification is a standard task in computer vision. Given an input image and a set of class labels, the classification algorithm outputs the most probable label (or a probability distribution over all the labels) for the image. Object classifiers are limited to categorizing a single object per image. Object detectors can locate and classify multiple objects in a given scene. The deep-learning-method-based object detectors can be classified into two categories: two-stage strategy detectors, such as FastRCNN [55], RCNN [56], SPPNet [57], Faster RCNN [41], R-fcn [58], MaskRCNN [59], and ResNet [44], and one-stage detectors, including DetectorNet [60], OverFeat [61], YOLO [40], and DETR [42]. Below, we detail DETR, YOLO, and Faster RCNN from the above two categories.

For DETR and YOLO, one-stage region-based frameworks, class probabilities and bounding box offsets are predicted directly with a single feedforward CNN network. This architecture leads to a faster processing speed. Due to such excellent efficiency and high-level accuracy, YOLO and DETR are good choices in real-time processing systems, such as the traffic light detection module in Apollo (an open platform for autonomous driving) and the object detection module in satellite imagery.

Faster RCNN, a two-stage detection framework, includes a preprocessing step for region proposals and a category-specific classification step to determine the category labels of the proposals. Faster RCNN is proposed to improve the RCNN, which is quite computationally expensive despite its high object detection accuracy. Instead of using the time-consuming selective search algorithm on the feature map to identify the region proposals, Faster RCNN uses a separate network to make the region proposals. Hence, Faster RCNN is much faster than its predecessors and can even be used for real-time object detection.

### 3.2. Attack Formulation

**Attacker’s Objective:** The attack is focused on object detection in image frames. We use $X\in R^{W\times H\times C}$ to represent the distribution of images, where each image (x∈X) has a width of *W*, a height of *H*, and a number of channels of *C*. We define Y={0,1,···,N−1} as the label space, where the number of classes is *N*. We use F(x):X→Y to describe the model inference that takes an image (x∈X) as input and forecasts the class label as y∈Y. In this paper, we focus on the hostile patch attacks against image detection models. Formally, given a deep-learning- or transformer-based model (*f*), an image (*x*), and its true class label (*y*), the goal of the attacker is to find an image ($x^{\prime }\in A\left(x\right)\subset X$) such that $f\left(x^{\prime }\right)=y^{\prime }$, where $y^{\prime }$ is an unreasoned class label defined by the attacker, and $y^{\prime }\neq y$.

**Attacker’s Capability:** The attacker can indiscriminately modify pixels within a confined region, which can be located anywhere in the image, including over the salient object. We assume that all the manipulated pixels are within the image frame region. Officially, we assume the adversary can arbitrarily reform an image (*x*) within a constraint set (A(x)). We use a binary pixel block (*p*) to denote the restricted region and $p\in P\subset {\left\{0,1\right\}}^{W\times H}$, where the pixels within the hostile division are set at 1. Then, the constraint set (A(x)) can be expressed as $x^{\prime }=\{\left(1-p\right)⊙x+p⊙x^{\prime \prime }\left|x,x^{\prime }\in X,x^{\prime \prime }\in R^{W\times H\times C},p\in P\right\}$, where ⊙ refers to the element-wise product operator, and $x^{\prime \prime }$ is the content of the adversarial patch.

## 4. “Detect-And-Inpaint” Strategy-Based Robust Vision Framework

**Figure 1:**
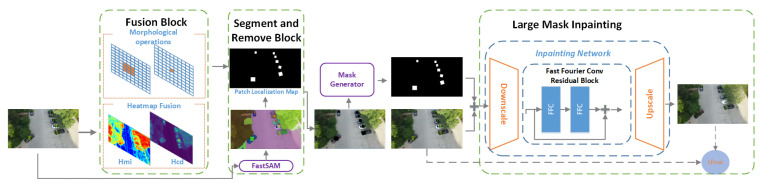
Segment-and-Recover (SAR) patch-agnostic defense framework.

In this section, we introduce the patch defense pipeline based on image preprocessing, as shown in Figure 1. For patch attacks in the physical scenes, we aim to eliminate the visible adversarial noises from examples by processing the inputs from the perspectives of the appearance inconsistency and adversarial attack effect. The input processed by our method can enable the subsequent classification model or detection model to make correct inferences. Our proposed pipeline can achieve a universal defense against adversarial patch attacks. Unlike PAD as the baseline framework, our modified and enhanced framework is constructed of a patch-region-localizing (frontend) and an inpainting (backend) pipeline. The localizing module aims to localize and segment the patch areas. The inpainting module performs the lost pixel recovery and adversarial perturbation elimination of the located possible patch areas. Instead of employing a simple and fast inpainting method that is commonly used in previous works, such as DW [17], SentiNet [18], SAC [19], PAD [20], and PatchZero [21], filling the patch area with all black pixels, our backend outperforms to guarantee the accuracies of the detection and classification when the patch occludes the object.

### 4.1. Patch Localizing and Feature Extraction (Frontend)

In our framework, we use FastSAM to segment the patch regions. Due to the limitations of the recognition capabilities of FastSAM, an extra prompt algorithm is needed to identify the adversarial patch among random segmentations. Since adversarial perturbations are often subtle and high frequency, we identify regions that have been digitally hacked or that have higher frequencies compared to those of adjacent regions. The patch prompt method is on lines 13–23 of Algorithm 1. Using a compression algorithm, such as JPEG, the pixel values are converted to the frequency domain using a discrete cosine transform (DCT). The distributions of DCT coefficients in authentic and adversarial-patch-modified regions will differ.
**Algorithm 1** Segment and Recover (SAR)**Input:** 
input image *x*, window size $\left(w_{x},w_{y}\right)$, matching threshold *T*, Base Detector BaseDetector(·).**Output:** 
robust detection $D^{*}$, inpainted images and CAUTION  1:**procedure** Sar$\left(x,w_{x},w_{y},T\right)$  2:am← AdvPredictor$\left(x,w_{x},w_{y},T\right)$  3:pm← AdvDetector(x,am)  4:$x^{\prime }\leftarrow$ PixelRestoration(x,pm)  5:D← BaseDetector$\left(x^{\prime }\right)$  6:**if** a==True **then**  7:      $D^{*}\leftarrow CAUTION$  8:**else**  9:      $D^{*}\leftarrow D$10:**end if**11:**return** $D^{*}$12:**end procedure**   13:**procedure** AdvPredictor$\left(x,w_{x},w_{y},T\right)$14:fm ← Fe(*x*)15:X,Y,_ ← Shape(*fm*)16:am ← ZeroArray[*X*, *Y*, *N* + 1]17:**for** each valid (i,j) **do**18:      l,v← Jpeg$\left(fm\left[i:i+w_{x}:j+w_{y}\right]\right)$19:      $am\left[i:i+w_{x},j:j+w_{y}\right]\leftarrow am\left[i:i+w_{x},j:j+w_{y}\right]+v$20:**end for**21:am ← Binarize(*am*, *T*, *w_x_*, *w_y_*)22:**return** am23:**end procedure**  ▹ Adversary detection (frontend) ▹ Adversary localization
▹ Broken pixel restoration (backend)
▹ Conventional detection 
▹ Trigger a caution      
▹ Extract feature map
▹ Get the shape of **fm**
▹ Initialization
▹ Every window location   ▹ Binarization    24:**procedure** AdvDetector(x,am)25:seg ← FastSamAutomaticMaskGenerator(*x*)26:L,T,R,B ← Shape(*seg*, *am*)27:$A_{box1}$ ← Area_Seg$\left[L_{1},T_{1},R_{1},B_{1}\right]$28:$A_{box2}$ ← Area_Am$\left[L_{2},T_{2},R_{2},B_{2}\right]$29:**for** each valid $A_{box1}$ **do**30:      **for** each valid $A_{box2}$ **do**31:        $A_{inter}\leftarrow \left(R_{inter}-L_{inter}\right)\times \left(B_{inter}-T_{inter}\right)$32:        $A_{union}\leftarrow A_{box1}+A_{box2}-A_{inter}$33:        $IoU\leftarrow \frac{A_{inter}}{A_{union}}$34:        **if** IoU<0.9 **then**35:             **return** 036:        **else**37:             pm ← Binarize(*am*)38:             **return** pm39:        **end if**40:    **end for**41:**end for**42:**end procedure**▹ Extract segmentation layer by layer ▹Get the left, top, right, and bottom of **seg** ▹ Initialization area of segmentation ▹ Initialization area of adversarial patch map ▹ Every segmentation ▹ Every Am ▹ Area of intersection ▹ Area of union ▹ Calculate IoU ▹ No overlap ▹ Binarization ▹ Return patch map    43:**procedure** PixelRestoration(x,pm)44:**while** i≤T **and** n>0 **do**45:      $C^{H\times \frac{W}{2}\times 2C}\leftarrow R^{H\times W\times C}$46:      $R^{H\times \frac{W}{2}\times 2C}\leftarrow C^{H\times \frac{W}{2}\times C}$47:      $R^{H\times \frac{W}{2}\times 2C}\leftarrow R^{H\times \frac{W}{2}\times 2C}$48:      $C^{H\times \frac{W}{2}\times 2C}\leftarrow R^{H\times \frac{W}{2}\times C}$49:      $R^{H\times W\times C}\leftarrow C^{H\times \frac{W}{2}\times C}$50:**end while**51:**return** $x^{\prime }$52:**end procedure**   ▹ Apply real FFT to input tensor ▹ Concatenate real and imaginary parts ▹ Apply a convolution block in the frequency domain  ▹ Apply an inverse transform to recover a spatial structure   


Since high frequencies are often removed in JPEG images by setting the respective DCT-coefficients at 0 in patches of 8×8 pixels, we use JPEG compression [62] to isolate the patch-represented high-frequency area. When the image is recompressed using the JPEG compression standard, the DCT coefficients are converted in compression and then processed using quantization. By applying quantization, the coefficients representing the high frequencies are set at zero, causing the high frequencies to disappear from the image. The quantization process can be expressed as follows:

(1) $$F_{Q}\left(x,y,i\right)=round\left(\frac{F(x,y,i)}{Q(x,y,i)}\right)$$

where Q(x,y,i) represents the corresponding quantization step size. F(x,y,i) represents the DCT coefficient of channel *i* at location (x,y). We quantify the pixel values before and after recompression as follows:

(2) $$H_{cd}\left(x,y,Q_{r}\right)=\frac{1}{c}\sum_{i=1}^{c}{\left[F_{Q_{c}}\left(x,y,i\right)-F_{Q_{r}}\left(x,y,i\right)\right]}^{2}$$

where *c* denotes the number of channels, and $Q_{c}$ and $Q_{r}$ represent the quality factors of the clean and adversarial-patch-attacked images. By subtracting the original images from a compressed image, only the high frequencies of an image are isolated, which aids in identifying real-world patches.

We perform a texture analysis by calculating the dissimilarity texture feature to gauge the homogeneity of pixel intensities in an image. High values indicate noisy textures, while low values indicate flat textures. To assess the local difference of the patch-attacked area in the texture of an image, a sliding window is built to capture the texture properties. The local mutual information heat map ($H_{mi}$) within the current window ($W_{cur}$) can be expressed as the average of the mutual information between $W_{cur}$ and its neighboring $W_{i}$ as follows:

(3) $$\begin{array}{c} H_{mi}\left[x_{cur}:x_{cur}+d,y_{cur}:y_{cur}+d\right]=\frac{1}{n}\sum_{i=1}^{n}\sum_{w_{i}\in W_{i}}\sum_{w_{c}\in W_{cur}}p\left(w_{i},w_{c}\right)log\frac{p(w_{i},w_{c})}{p\left(w_{i}\right)p\left(w_{c}\right)} \end{array}$$

We fuse the normalized local mutual information heat map ($H_{mi}$) and the recompression difference heat map ($H_{cd}$) as follows:

(4) $$H\left(x,y\right)=r_{mi}\times H_{mi}+\left(1-r_{mi}\right)\times H_{cd}$$

where $r_{mi}$ denotes the weight of the mutual information heat map. We set up $r_{mi}$ to be 0.5.

The second stage is detecting and segmenting all the objects, including the adversarial patches, that impact the inference accuracy of object classification and detection in the image. FastSAM [63], a unified model, achieves a high-precision, class-agnostic segmentation for video and image segmentation, exhibiting distinct capabilities in zero-shot tasks. Those visible perturbations could be any shape, scale, or location. This stage separates the specific object(s) of interest from the segmented panorama, which depends on the provided prompts. These prompts range from foreground/background point sets to rough boxes or masks, free-form text, or any information that indicates the content to be segmented within an image. However, the location, scale, and shape of adversarial perturbations are unknown beforehand. So, we only rely on FastSAM’s zero-shot segmentation capability to isolate the edges of all the regions in the patch-attacked images and obtain masks for adversarial patches. We then match each mask with $H_{p}$ and consider all the masks with an intersection-over-union (IoU) value of greater than the threshold as the final patch masks on lines 24–42 of Algorithm 1. The equation for the IoU is given as follows:

(5) $$IoU\left(mask,H_{p}\right)=\frac{area(mask⋂H_{p})}{area(mask)}$$

### 4.2. Patch Mask Inpainting with Fourier Convolutions (Backend)

The detection accuracy often struggles with large missing areas of objects, complex geometric structures, and high-resolution images. Although [24,25,26,27] propose to remove and refill the identified candidate regions, using inpainting to mitigate the potential adverse effects, accurately determining the key adversarial pixel is unrealistic because the adversarial gradient of the patch is unknown beforehand. Furthermore, the refilled area will occlude the objects to be detected or classified because it contains too many pixels, which are far away from the original object, if the adversarial patch covers too much area of the object. To alleviate this issue, we develop a backend for large mask inpainting to restore the broken pixels. The restored pixels guarantee that a detector or a classifier can identify the patched–occluded object. The method on lines 43–52 of Algorithm 1 is to inpaint a color image (*x*) masked by a binary mask of unknown pixels (*m*), where the masked image is denoted as x⊙m. The mask (*m*) is assembled with the masked image (x⊙m), generating a four-channel input tensor ($x^{\prime }=stack\left(x⊙m,m\right)$). We use a preorder inpainting network ($f_{\theta }\left(\cdot \right)$), which we also refer to as the generator. Taking $x^{\prime }$, the inpainting network proceeds with the input in a fully convolutional manner and produces an inpainted three-channel color image ($\hat{x}=f_{\theta }\left(x^{\prime }\right)$). For wide masks, the whole receptive field of a generator at a specific position may be inside the mask, thus observing only missing pixels. We have a channel-wise fast Fourier transform (FFT)-based fast Fourier convolution (FFC) to generate the receptive field that covers the entire image. The FFC splits channels into local and global branches. The local branch conducts the conventional convolutions. The global branch implements the real FFT to account for the global context. We finally fuse the local and global branches together.

## 5. Evaluation Study

In this section, we evaluate the performance of the proposed SAR framework in standard benchmark datasets under a range of patch-based adversarial attacks. We further compare our method with state-of-the-art approaches using established evaluation metrics reported in the literature.

### 5.1. Metrics

#### 5.1.1. Clean Performance Metrics

Average Precision (AP) [64]: We measure the accuracy of the detector in identifying and classifying objects within an image to report the average precision. We modify the confidence threshold, from 0 to 1, to document the precision and recall at various thresholds and calculate mAP as the averaged precision at 0.5. This calculated AP can be considered as an approximation of the area under the curve (AUC) for precision–recall curves. We note that mAP is one of the most widely used performance metrics in object detection benchmark competitions and research papers.

False-Alert Rate (FAR) [65]: The FAR is determined as the ratio of clean images on which SAR will prompt a false caution when an adversary is available in image frames. The FAR is also intimately related to the confidence entry of the detector: A higher confidence threshold causes fewer anticipated bounding boxes, leading to more inexplicable objectness and, finally, a higher FAR.

#### 5.1.2. Provable Robustness Metrics

Patch Localization Recall (PLR) [66]: We use patch localization recall to evaluate the localization performance of the “detect-and-inpaint” strategy. It represents the percentage of applied patches that have been detected by the detection-based frontend with an intersection-over-union value that exceeds 0.9.

Certified Recall (CR@0.5) Values [36]: We use certified recall as the robustness metric against patch-hiding attacks. The certified recall is defined as the percentage of ground-truth objects that have provable robustness against any patch-hiding attack. Recall that an object has provable robustness when Algorithm 1 identifies the objects from an adversary.

### 5.2. Evaluation Setup and Benchmarks

Adversarial patch attacks: To assess the defense performance of SAR against diverse types of patches, we employ nine varied patches generated by EAVISE [47], DPatch [1], and the YOLO adversarial patch [48], representing the localized-noise, printable, and object-occluded patches. DPatch produces constraint-sized patches (40 mm × 40 mm, 75 mm × 75 mm, and 100 mm × 100 mm) placed in the upper left corner of each image. The YOLO adversarial patch, covering (20%,30%, and 40%) of the bounding box, is placed over the target objects. The bounding boxes are generated by running the same detector over the dataset. EAVISE creates a small patch (around 40 mm × 40 mm) that is used to hide people from object detectors, where the objective was to minimize the products of the object score and the class score (OBJ-CLS), only the object score (OBJ), and only the class score (CLS).

Dataset and Architecture: We apply the adversarial patch on the Visdrone dataset [67] to generate the patch-attacked image subset. We create a class of 400 adversarial-patch-attacked images as a subset from the original set. Those adversarial patches are overlaid at the target object detection location and do not fully occlude the target object, only covering 10% to 30% of the bounding box’s size. The adversarial goal is to evade the detection of target objects.

Detection tasks: For YOLO [40], a one-stage region-based framework, class probabilities and bounding box offsets are predicted directly with a single feedforward CNN network. This architecture leads to a faster processing speed. DETR [42], which stands for detection transformer, is a deep learning model that utilizes a transformer encoder–decoder architecture. It is known for streamlining the object detection pipeline by directly predicting a set of objects without relying on many handcrafted components, like non-maximum suppression or anchor boxes. Faster RCNN [41], a two-stage detection framework, includes a preprocessing step for region proposals and a category-specific classification step to determine the category labels of the proposals. These detector models are pretrained on MS COCO [68].

Benchmarks: We compare SAR with four state-of-the-art adversarial patch defenses: LGS [24], Jedi [28], RLID [29], and Jujutsu [25], corresponding to “detect-and-mitigate”, “detect-and-recover”, and “detect-and-remove” strategies.

### 5.3. Results: Clean Performance

**Figure 2:**
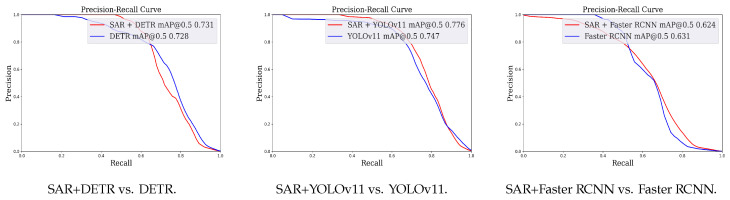
PR curves of SAR defenses with various detectors in the PASCAL VOC [69] dataset.

In this section, we evaluate the clean performance of SAR with three different base object detectors and three datasets. In Table 3, we evaluate the AP and FAR in a clean recall. Also, we plot the precision–recall curves, shown in Figure 2, for the PASCAL VOC [69] challenge dataset.

SAR has a low FAR and a high AP. Table 3 shows that when SAR uses various clean detectors as its base, it achieves a very low false-alarm rate (FAR) of just 0.2% and maintains high average precision (AP). These results indicate that SAR has only a negligible effect on clean detection performance. In fact, its mean average precision (mAP) is nearly identical to those of the base detectors.

SAR is greatly compatible with various object detectors. We report the mAPs of clean samples, after the defense, in Table 3. We can conclude that when we use DETR, YOLOv11, or Faster R-CNN as base detectors on diverse patches, the clean AP, as well as the precision–recall curves of the SAR, as shown in Figure 2, is close to that of its base detector (with a 1.1% drop on Faster R-CNN, a 3.7% rise on YOLOv11, and a 0.4% rise on DETR). These results show that SAR is greatly functional with various object detectors.

### 5.4. Results: Provable Robustness

In this subsection, we present the robustness evaluation studies and manifest the provable robustness of the defense, SAR, against any hostile patch attack.

#### 5.4.1. Patch Localization Performance

Patch localization is a critical step in the detection frontend defense pipeline, as it forms the foundation for restoring the broken pixels. We compare our “detect-and-inpaint” approach alongside PAD [20], SentiNet [18], and Jujutsu [25]. All four use patch detection as their frontend. In our experiments, where patches could appear anywhere on the object or in the image, our method achieved the highest patch localization recall rate (see Table 4). This observation demonstrates that our method can accurately detect potential risks from images, especially when the adversary conceals the object from detection. Admittedly, masking a larger image area can slightly reduce the object detection mAP, but our method consistently delivers the most accurate localization among preprocessing frontends.

#### 5.4.2. Defense Against Adaptive Patches in Object Tracking

**Figure 3:**
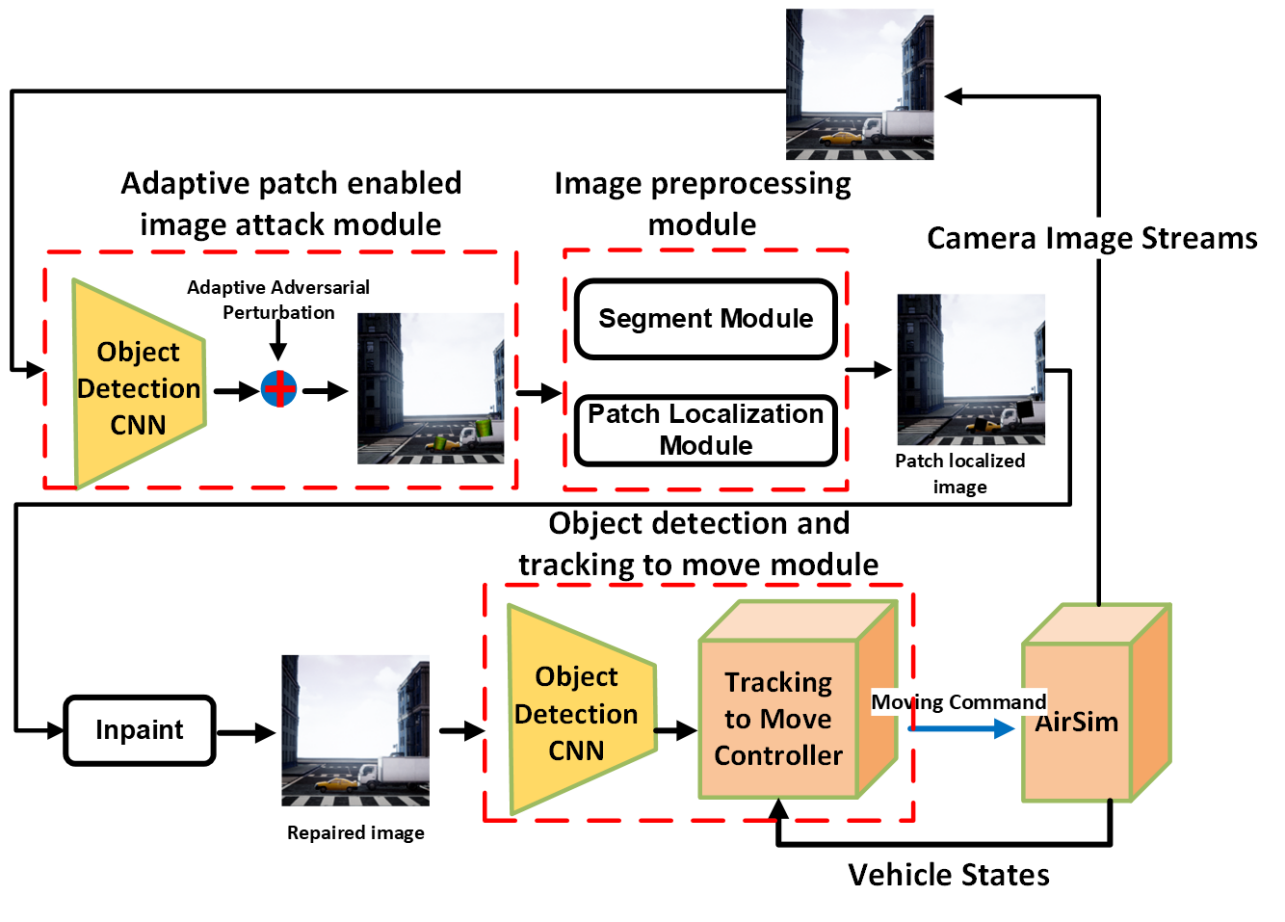
Using the AirSim simulation environment [72], we simulated a scenario in which a quadrotor drone tracks a car in an urban setting, using its onboard camera. The YOLO model [40] is used to process the image frame and localize the car with respect to the drone, which the tracking controller then uses to move the drone. In our simulations, the vision-based object tracking is subject to adaptive patch attacks, where we overlay an adversarial patch on the target to mislead the victim object detector within the vision-based tracking system. Lastly, we apply SAR to preprocess the attacked-image frames.

We further evaluate the defense performance under adaptive patch attacks, where the adversary targets the detector in the vision-based object-tracking pipeline shown in Figure 3. Using the AirSim simulation environment [72], we simulated a scenario in which a quadrotor drone tracks a car in an urban setting, using its onboard camera. The YOLO model [40] is used to process the image frame and localize the car with respect to the drone, which the tracking controller then uses to move the drone. To perform the adaptive attack, we first use YOLO to generate bounding boxes for each image frame. Then we place adversarial patches, crafted by the AD_YOLO method [48], on the detected objects at three different patch sizes.

From Table 3, SAR demonstrates strong provable robustness when targets are covered with patches at various scales. This result indicates that SAR significantly increases the difficulty of successful adversarial attacks: To bypass SAR, the adversary must place the patch directly on the target object. We also note that in our threat model, the patch is allowed to appear anywhere on the object, including in its most salient regions. Patches covering such critical features typically make robust detection extremely challenging. Nonetheless, SAR remains highly effective, even when the patch obscures the most informative part of the object. Overall, SAR achieves the strongest robustness among all five baselines under these adaptive, scale-varying, and strategically placed patch attacks.

#### 5.4.3. Defense Against Physical (Printable) Patch Attacks

We further validate the effectiveness of SAR against a wider range of patch types, specifically physical (printable) patches. The printable patches include three adversary tasks: OBJ, OBJ-CLS, and CLS. We evaluate the certified recall at a clean recall of 0.5 in Table 3. Provable robustness boosts as clean recall exaggerates, and the performances of DETR, YOLOv11, and Faster R-CNN approach that of an ideal clean detector when the recall is close to one. As shown in Table 3, when using a perfect clean detector, SAR can certify the robustness for 71% of the objects when under a patch attack (for various adversary tasks), meaning that none of the attackers within our hostile model can effectively attack these certified objects.

#### 5.4.4. Defense Against Localized Patch Attacks

The defense performance against localized-noise attacks (DPatch [1]) is shown in Table 3, where the attacker targets only the object detectors. The localized noise encompasses three different patch sizes. As shown in Table 3, SAR is robust across different patch sizes in both datasets and has the highest (CR@0.5) value compared to those of the baselines. In addition, adversarial patches create counterfeit detections and vague foreground objects at diverse scales. SAR masks out adversarial patches and restores model predictions. These results manifest that SAR is a generalizable method that can be applied to easier or more challenging detection tasks.

### 5.5. Ablation Study

**Figure 4:**
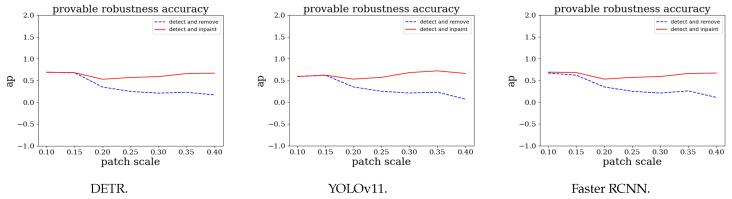
Provable robustness accuracies of defenses with different detectors in the patch-attacked Visdrone dataset [67].

We perform an ablation study to investigate the individual impacts of the image-inpainting backend. We first conduct a runtime analysis of SAR to manifest its lightweight overhead. Next, we compare the AP in the “detect-and-inpaint” and “detect-and-remove” strategies in Figure 4.

Real-time Test: We tested the real-time performance of the “detection-and-inpaint” pipeline within a vision-based tracking-to-movement system, as shown in Figure 3. In this setup, an adversarial patch attacks targets in the tracking system: Patches of sizes 30%, 35%, and 40% of the bounding box are placed over the tracking target (e.g., cars) to prevent detection. As the tracking target moves in the image frame, the overlaid patch location and scale change from frame to frame, and, hence, we consider it to be an “adaptive” attack. We embed the “detect-and-inpaint” pipeline to preprocess the adversarial image frame to robustify the system. Considering only one available GPU, the SAR runtime can be calculated as $t_{predictor}+t_{detector}+t_{inpaint}$, which achieves the real-time image restoration performance in Table 5. Overall, SAR leads to a roughly 2× slowdown compared to that of an undefended detector.

The Effect of a Broken Pixel Restoration Backend: We calculate the APs for popular detectors in a subset of the VisDrone dataset [67]. As shown in Figure 4, when using the pixel restoration backend, the detector is able to identify objects, even when adversarial patches cover between 10% and 40% of the detection-bounding box. The figure also shows that AP improves as the broken pixels are restored, with the performances of DETR, YOLOv11, and Faster R-CNN approaching that of an exemplary clean detector. As a result, the pixel-recovering process minimizes the adverse effects. Table 1 presents the object detection results obtained using DETR, YOLOv11, and Faster R-CNN. When adversarial images are preprocessed with the “detect-and-remove” strategy, the black mask used to cover the patch can obscure the object, leading to detection failure due to excessive pixel loss. In contrast, when using the inpainting module to repair the corrupted regions, the objects are accurately detected, as shown in Table 1.

## 6. Generalization and Limitations

**Figure 5:**
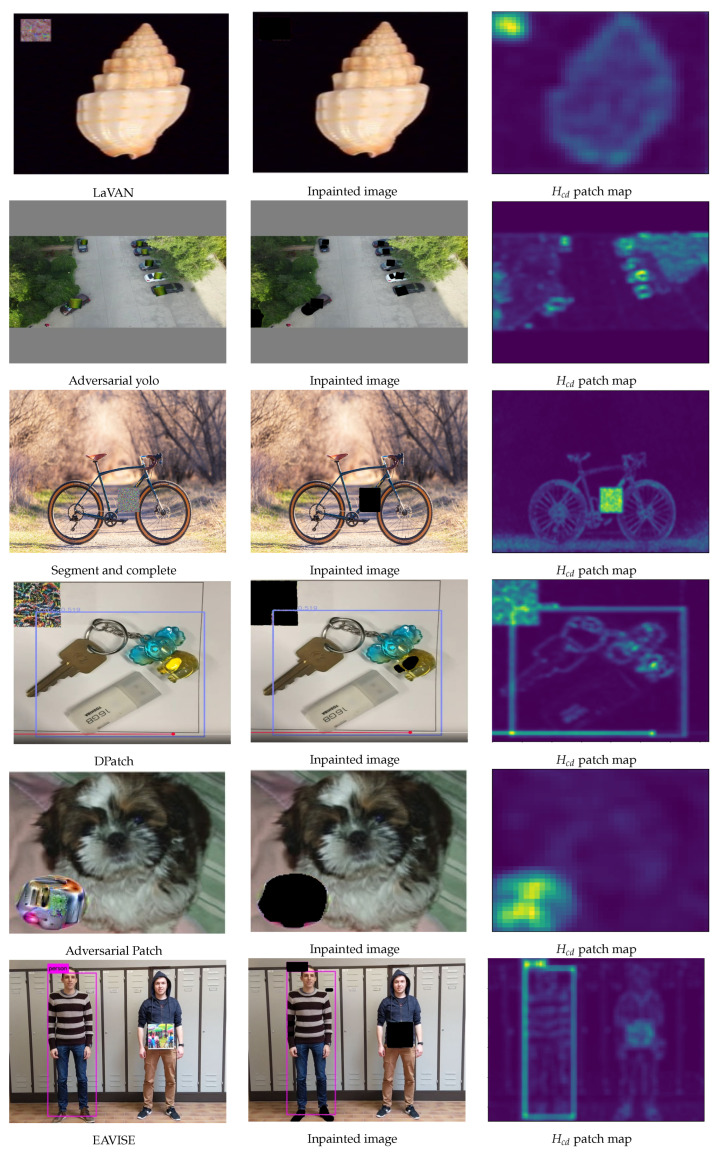
Visualization examples illustrating the patch localization process across different adversarial patch types. The highlighted yellow in third column denote the adversarial pixel.

**Figure 6:**
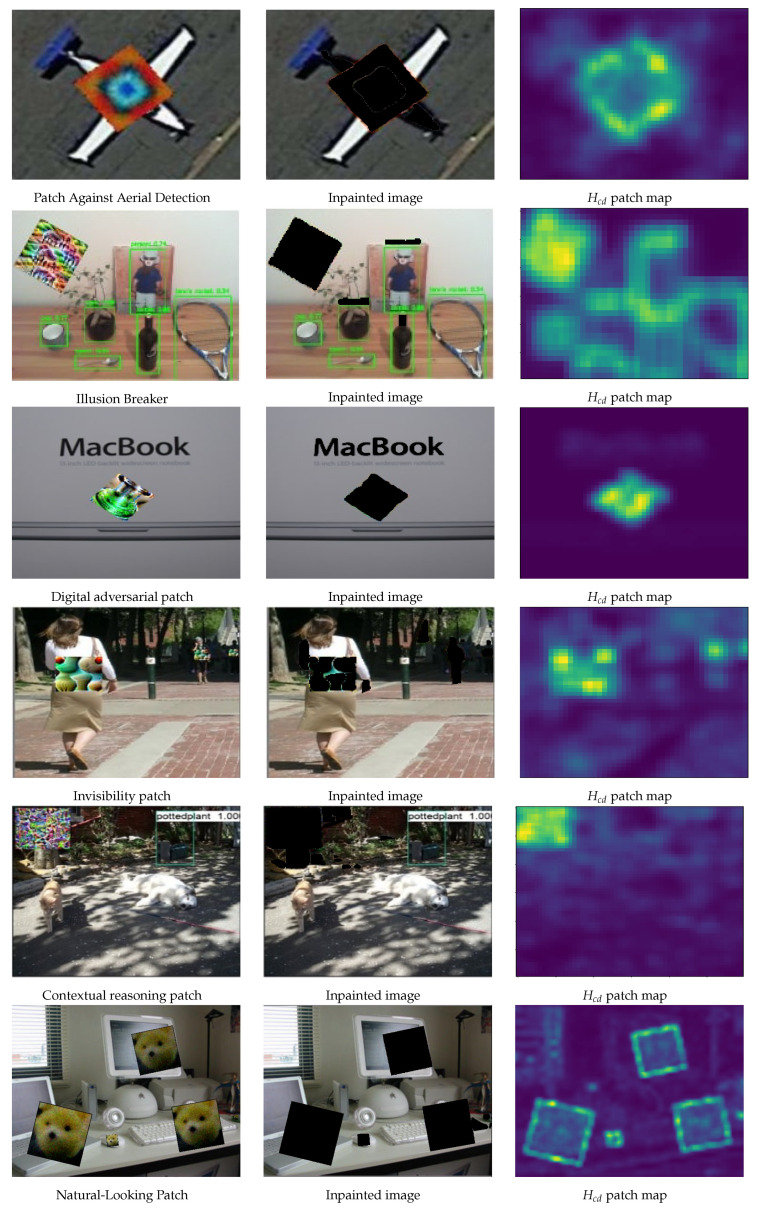
Visualization examples illustrating the patch localization process across different adversarial patch types. The highlighted yellow in third column denote the adversarial pixel.

Generalization to Various Adversarial Patches: Since adversarial patches may not always be square in the real world, we further evaluate SAR with adversarial patches of diverse shapes while varying the number of pixels in the patch. In this paper, we used the following state-of-the-art adversarial patches for a comprehensive evaluation: LaVAN [11], Adversarial yolo [48], Adversarial Patch [9], DPatch [1], EAVISE [47], DPatch [1], Extended RP_2 [2], Physical patch + PGD [73], the Patch-Against-Person Detector [47], DPAttack [3], RPAttack [4], the Patch Against Aerial Detection [5], Patch Noobj [6], Translucent Patch [50], Dynamic Patch [74], Invisibility Patch [75], the Patch Exploiting Contextual Reasoning [49], Illusion Breaker [76], and the Natural-Looking Patch [77]. Each of the patch categories is a unique attack scheme, and these patches represent the majority of the existing visible adversarial patch schemes, to the best of our knowledge. The results are shown in Figure 5 and Figure 6. SAR demonstrates strong robustness under circular, rectangular, triangular, diamond-shaped, and ellipsoidal patch attacks.

Limitations: The proposed defense is effective to protect against localized and visible attacks in its current form; however, we have shown that the Translucent Patch [50] allows attackers to bypass the defense. This highlights the need for further research on how best to defend against such attacks. One promising direction is to exploit the translucent structures of images in contrast to the unnatural structures of adversarial perturbations. For example, we observed empirically that most salient objects in ImageNet samples contain thin, continuous regions, representing the most influential image areas, whereas our modified attack generates sparse, noisy patterns. However, additional research is needed to determine whether this kind of structural reasoning can be generalized to defend against localized and visible adversarial perturbations in other domains.

## 7. Conclusions

In this paper, we identify frequency domain characteristics of adversarial patches that are independent of their appearance, shape, scale, and location. Leveraging these characteristics, we propose a generalized patch-agnostic defense method, SAR, which performs adversarial patch localization and restoration against patch-hiding attacks. SAR adapts robust image classifiers for robust object detection using an objectness-explaining strategy. Our evaluation of the different datasets demonstrates that SAR outperforms the defense approaches evaluated in this paper against patch-hiding attacks and exhibits a high degree of compatibility, with a clean performance comparable to those of state-of-the-art object detectors.

## Figures and Tables

**Table 1:** **(Top Row)** Due to the added patches, the PAD [20] (baseline approach) yields significantly degraded inference accuracy when evaluated using three different object detection models: YOLOv11 [40], Faster RCNN [41], and DETER [42]. **(Bottom Row)** The inference results of SAR (our approach), which present the detection performance with a high level of confidence.

	YOLOv11x [40]	Faster RCNN [41]	DETR [42]
PAD	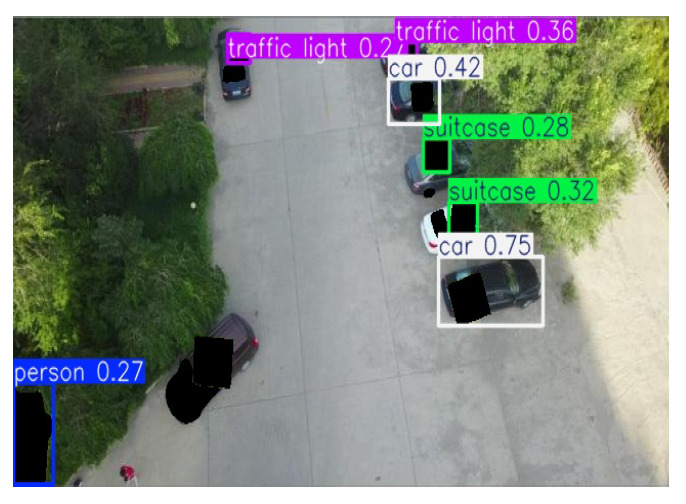	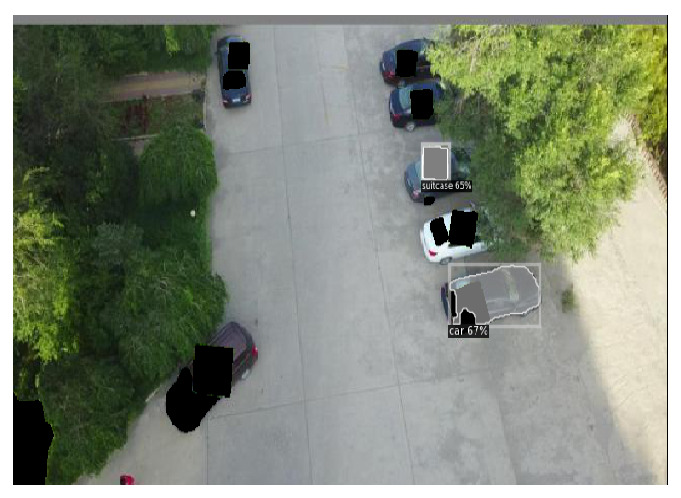	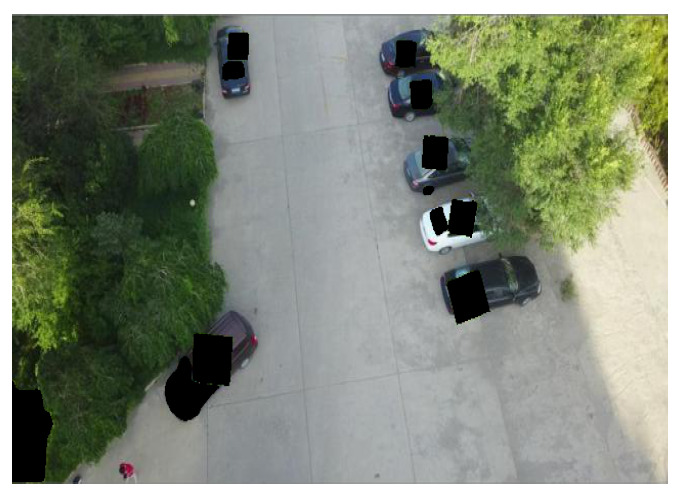
SAR	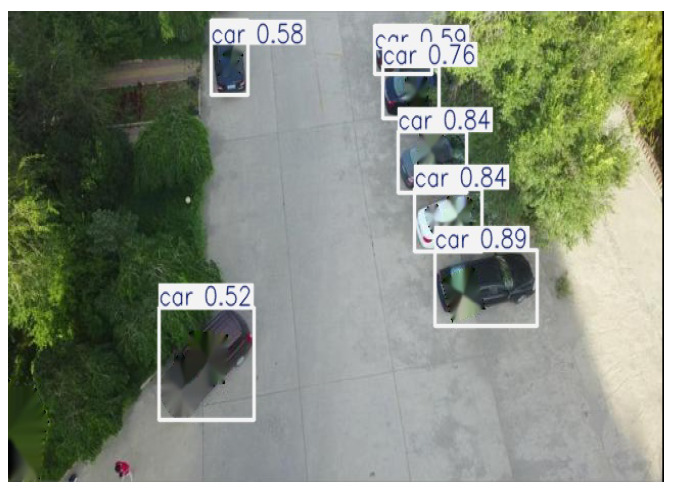	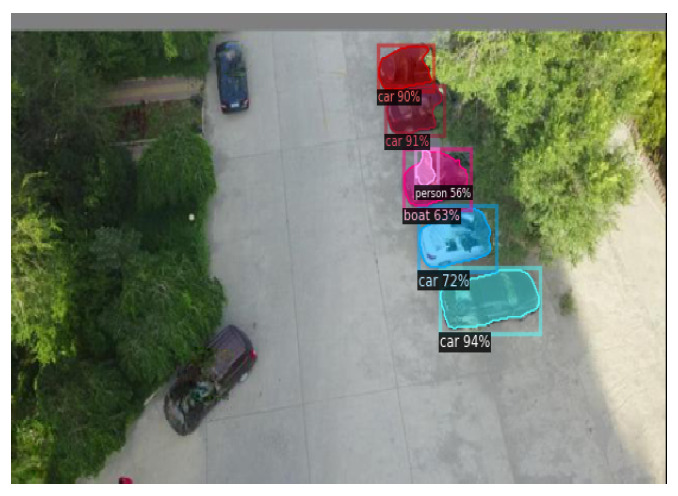	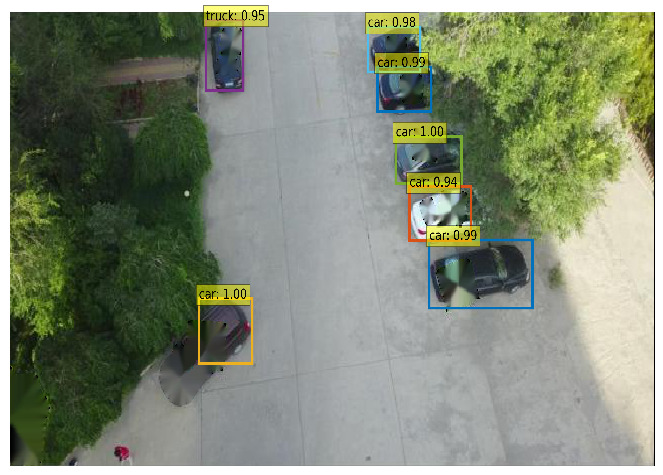

**Table 2:** Table of notations.

Notation	Description
$X\subset R^{W\times H\times C}$	image space
Y={0,1,···,N−1}	label space
M(x):X→Y	model predictor from x∈X
A(x)	constraint set
$p\in P\subset {\left\{0,1\right\}}^{W\times H}$	binary pixel block
am	adversarial patch mask map

**Table 3:** CR@0.5 values under various hostile patch attacks. The bold numbers highlight the best performances.

Detector	Defense	Clean	FAR	Printable Patch (EAVISE [47])			Localized Noise (DPatch [1])			Adaptive Patch (Ad_yolo [48])		
				OBJ-CLS	OBJ	CLS	40×40	75×75	100×100	20%	30%	40%
	Undefended	0.66	n/a	0.53	0.57	0.61	0.55	0.39	0.55	0.30	0.53	0.36
	LGS [24]	0.55	0.083	0.42	0.56	0.60	0.32	0.33	0.57	0.43	0.35	0.25
DETR	Jedi [28]	0.39	0.07	0.62	0.58	0.48	0.55	0.39	0.55	0.61	0.46	0.35
	RLID [29]	0.62	0.022	0.54	0.69	0.38	0.69	0.68	0.41	0.46	0.52	0.55
	Jujutsu [25]	0.58	0.068	0.62	0.58	0.68	0.32	0.33	0.57	0.43	0.35	0.25
	SAR (Ours)	**0.70**	**0.002**	**0.82**	**0.86**	**0.70**	**0.69**	**0.68**	**0.74**	**0.66**	**0.62**	**0.75**
	Undefended	0.57	n/a	0.61	0.46	0.35	0.35	0.51	0.49	0.64	0.69	0.58
	LGS [24]	0.71	0.019	0.33	0.25	0.34	0.37	0.32	0.35	0.59	0.70	0.62
YOLOv11	Jedi [28]	0.62	0.021	0.35	0.44	0.26	0.35	0.49	0.37	0.68	0.34	0.61
	RLID [29]	0.61	0.07	0.46	0.62	0.55	0.49	0.68	0.55	0.64	0.69	0.58
	Jujutsu [25]	0.59	0.065	0.43	0.35	0.25	0.32	0.33	0.57	0.62	0.58	0.68
	SAR (Ours)	**0.74**	**0.003**	**0.75**	**0.66**	**0.61**	**0.55**	**0.71**	**0.74**	**0.72**	**0.76**	**0.70**
	Undefended	0.61	n/a	0.35	0.51	0.49	0.30	0.53	0.36	0.53	0.57	0.61
	LGS [24]	0.57	0.083	0.37	0.32	0.35	0.66	0.46	0.55	0.54	0.69	0.70
Faster	Jedi [28]	0.35	0.022	0.35	0.49	0.37	0.35	0.44	0.26	0.68	0.74	0.61
R-CNN	RLID [29]	0.57	0.316	0.29	0.68	0.44	0.33	0.25	0.34	0.59	0.78	0.62
	Jujutsu [25]	0.62	0.019	0.32	0.33	0.57	0.43	0.35	0.25	0.62	0.58	0.68
	SAR (Ours)	**0.65**	**0.006**	**0.65**	**0.69**	**0.75**	**0.71**	**0.62**	**0.55**	**0.82**	**0.86**	**0.88**

**Table 4:** Patch localization recall (%). The bold numbers highlight the best performances.

	Defense	SAR	PAD	SentiNet	Jujutsu
Patch + Dataset					
LaVAN [11]	ImageNet [70]	**87.2**	44.5	39.60	12.17
DPatch [1]	Pascal VOC [69]	**91.47**	39.60	26.94	28.03
EAVISE [47]	Inria [71]	**74.20**	28.03	35.02	27.08
Ad_yolo [48]	VisDrone-2019 [67]	**93.29**	10.85	19.22	34.30

**Table 5:** Per-example runtime division.

	Base Detector	Objectness	Objectness	Inpaint	SAR
	yolov11	Predictor	Detector	Lama	yolov11
Small	69.0 ms	0.2 ms	0.55 ms	54.2 ms	54.95 ms
Medium	32.2 ms	0.4 ms	0.25 ms	56.5 ms	57.15 ms
Large	54.8 ms	0.3 ms	0.35 ms	56.2 ms	56.85 ms

## Data Availability

The original data presented in the study are openly available in https://github.com/robotics-star/SAR (accessed on 10 September 2025).
